# Significance of the ARIMA epidemiological modeling to predict the rate of HIV and AIDS in the Kumba Health District of Cameroon

**DOI:** 10.3389/fpubh.2025.1526454

**Published:** 2025-02-24

**Authors:** Arrey Emmanuel Besong, Odette Dzemo Kibu, Elvis Asangbeng Tanue, Besong Agbor Obinkem, Ginyu Innocentia Kwalar, Fabrice Chethkwo, Valentine Ndze Ngum, Maurice Marcel Sandeu, Patrick Jolly Ngono Ema, Nkweteyim Denis, Onduo Moise, Ayana Gelan, Jude Dzevela Kong, Dickson Shey Nsagha

**Affiliations:** ^1^Department of Public Health and Hygiene, Faculty of Health Sciences, University of Buea, Buea, Cameroon; ^2^Global South Artificial Intelligence for Pandemic and Epidemic Preparedness and Response Network (AI4PEP), Toronto, ON, Canada; ^3^Africa-Canada Artificial Intelligence & Data Innovation Consortium (ACADIC), Department of Mathematics & Statistics, York University, Toronto, ON, Canada; ^4^Digicare-Cameroon Consortium (DIGICARE-CAMEROON), Buea, Cameroon; ^5^School of Veterinary Medicine and Science University of Ngaoundere, Ngaoundere, Cameroon; ^6^Department of Computer Sciences, Faculty of Sciences, University of Buea, Buea, Cameroon; ^7^School of Biomedical Engineering, Jimma University, Jimma, Ethiopia

**Keywords:** ARIMA model, retrospective study, Kumba District Hospital, HIV/AIDS, Cameroon

## Abstract

**Background:**

AIDS is a severe medical condition caused by the human immunodeficiency virus (HIV) that primarily attacks the immune system, specifically CD4+ T lymphocytes (a type of white blood cell crucial for immune response), monocyte macrophages, and dendritic cells. This disease has significant health and socio-economic implications and is one of the primary causes of illness and death globally (UNAIDS, 2022). It presents significant challenges for public health and population well-being, both in developed and developing countries. By conducting a time series analysis, this research seeks to identify any significant changes in HIV rates over the next 4 years in the Kumba District Hospital and provide valuable insights to inform evidence-based decision-making and strategies for preventing and controlling HIV within the Kumba Health District.

**Materials and methods:**

A hospital-based retrospective study on HIV/AIDS recorded cases was conducted at the Kumba District Hospital. Using data extraction form, hospital records from 2012 to 2022 were reviewed and data extracted and used to make predictions on the number of future incidence cases. Time series analysis using Auto-Regressive Integrated Moving Average (ARIMA) model was done using Statistical Package for the Social Sciences (SPSS) Version 26.

**Results:**

According to the ARIMA parameter (p,d,q), the results for the Partial Autocorrelation Factor (p) was 1, differencing (d) was 0 and Autocorrelation Factor (q) was 0. Putting these values together, we had the ARIMA model (1,0,0) which predicted an overall increase in HIV incidence cases at the Kumba District Hospital for the upcoming Years (2023–2026).

**Interpretation:**

The ARIMA model was found to be independent of errors and a perfect fit, with a high R-squared value of 0.764 and a *p*-value of 0.410, indicating that the model’s predictions aligned well with the observed data. The model predicted an increase in the number of HIV incidence cases over the coming years (2023–2026), potentially suggesting a worsening situation. However, it is important to interpret these predictions with caution and consider other factors that may influence the incidence of HIV in reality.

## 1 Introduction

AIDS is a severe medical condition caused by the human immunodeficiency virus (HIV) that primarily attacks the immune system, specifically CD4+ T lymphocytes: which are a type of white blood cell that plays a crucial role in the immune response by helping to coordinate the activity of other immune cells monocyte macrophages, and dendritic cells (1). The global impact of HIV/AIDS is a significant concern, affecting individuals from all backgrounds and nations. Yet, it presents an even more significant challenge in underdeveloped countries, including countries within Sub-Saharan Africa (SSA), where over two-thirds of global infections are prevalent (2). Research indicates that following an HIV-positive diagnosis, it is crucial to ensure effective linkage to care (3). Currently, there is no cure for HIV/AIDS, and the only means of treatment to extend lifespan and enhance quality of life is through anti-retroviral therapy (ART) (4). The primary symptom of AIDS is a progressive loss of immune response, leaving the body vulnerable to opportunistic infections, tumors, and abnormalities (5). The Human Immunodeficiency Virus (HIV) is a highly infectious disease that has become a serious public health concern worldwide (WHO, 2022) (6). This disease has significant health and socio-economic implications and is one of the primary causes of illness and death globally (7). HIV-positive individuals, particularly those with AIDS, can transmit the virus to others through bodily fluids such as blood, semen, vaginal fluids, and breast milk, as well as through contaminated needles and syringes (8). Despite significant efforts, there are few effective drugs available for the treatment of HIV/AIDS, making it one of the leading causes of morbidity and mortality around the world (9). This presents enormous global challenges for public health and population wellness (10). According to According to the Joint United Nations Program on HIV/AIDS (UNAIDS) data from 2022, an average of 39 million people globally are currently living with HIV/AIDS and more than 600,000 people have already died from AIDS-related illnesses. In SSA countries, non-compliance to ART is prevalent, which is why the disease burden remains high within the region (11).

Cameroon, like most SSA countries, has experienced a rise in the number of new HIV cases. According to the National Institute of Statistics in Yaoundé, Cameroon, the prevalence of HIV among the Cameroonian population is 2.9%, with a higher prevalence among women (3.1%) compared to men (2.6%) (12). In 2022, an estimated 1.4% of pregnant women in Cameroon were living with HIV, and the vertical transmission rate was 1.2% (12). These data highlight the importance of targeted interventions, particularly for pregnant women and their partners, to prevent mother-to-child transmission of HIV. This places Cameroon among the countries in SSA with the highest death toll from HIV/AIDS (13). In the South West Region of Cameroon, the prevalence of HIV is estimated at 3.2% with urban areas being the most affected (14). Healthcare workers in this region have acknowledged that the ongoing political turmoil has drastically hindered the advancement of a successful response to the HIV/AIDS crisis in the Southwest region of Cameroon. Collaborative endeavors are required to fortify the healthcare sector in crucial aspects, including decentralizing HIV healthcare, enhancing the supply chain, and safeguarding healthcare workers from political entanglements. These initiatives are imperative to alleviate the effects of the social and political turmoil on the response to HIV/AIDS and the larger healthcare domain. Tracking and analyzing the rate of HIV over time is essential for effective public health planning, resource allocation, and implementation of targeted interventions. To date, it appears that no epidemiological studies have concentrated on projecting future occurrences of HIV infection within the Kumba Health District in the Southwest Region.

This study employs time-series modeling (Auto-Regressive Integrated Moving Average: ARIMA) using Kumba District Hospital HIV/AIDS data. With one decade of reliable data, this technique proves to be a viable approach to predict the incidence of health-related problems like HIV/AIDS. The methodology used in this research follows the Umunna (2020) approach, which used the Box-Jenkins forecasting method to predict new HIV cases in Mina Niger state, Nigeria (15).

The ARIMA methodology has been utilized in various studies to model epidemic incidence. One of these studies predicted yearly cases of Human Immunodeficiency Syndrome (HIV) using this methodology in Nairobi Kenya (16), while another study focused on forecasting monthly cases of HIV in the Philippines (17). These researchers used advanced statistical techniques to build the ARIMA model, employing the univariate Box-Jenkins method to forecast HIV cases. Furthermore, a study conducted by Abuya used a seasonal autoregressive integrated moving average (SARIMA) model to analyze the time series of malaria cases in the Kenya (18). Another study by Patel used a Bayesian vector autoregression (BVAR) model to analyze the time series of HIV/AIDS cases in South Africa (19). These studies demonstrate the effectiveness of time series models in analyzing the dynamics of infectious diseases and identifying trends and patterns that can inform public health policy.

By conducting a time series analysis, this research seeks to identify any significant changes in HIV rates over the next 4 years in the Kumba District Hospital, and provide valuable insights to inform evidence-based decision-making and strategies for preventing and controlling HIV within the Kumba Health District.

## 2 Materials and methods

### 2.1 Study area

**Figure 1:**
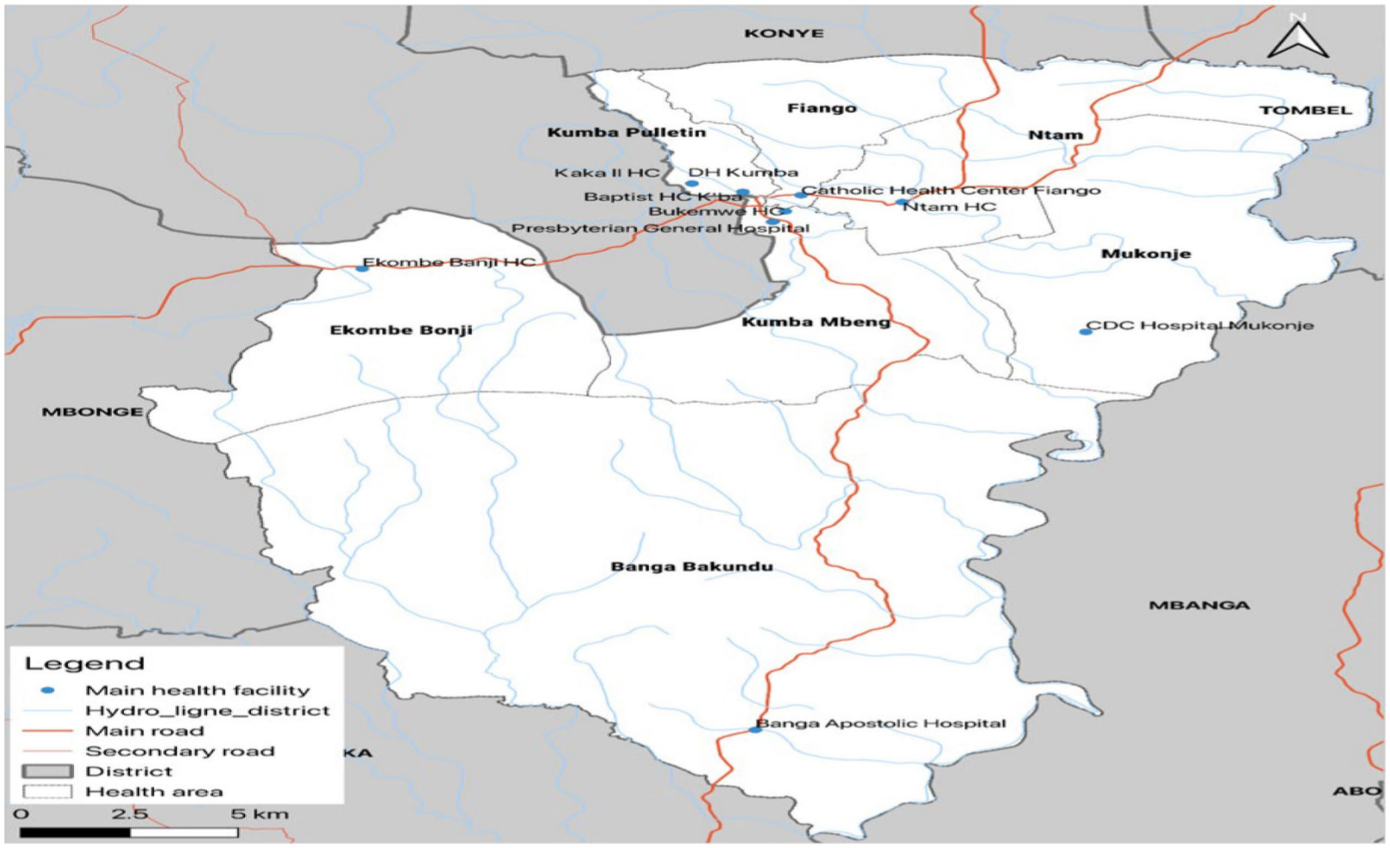
Map of Kumba health district.

The study area is located in the Kumba health districts, which are situated in the Meme Division of Cameroon. The Kumba health districts are served by two health facilities: Kumba-South and Kumba-North. Figure 1 shows Kumba Health district and its health areas. The study area spans from Kumba central sub-division, parts of the Mbonge sub-division, and parts of the Ndian division. It is bounded to the north by Konye Health District, to the west by Mbonge Health District, to the south by the Muyuka Health District, and to the east by the Littoral Region, whose capital is Douala. The Kumba District Hospital is one of the largest health facilities in the Meme Division of Cameroon and serves a population of approximately 265,071 inhabitants. The study area has a total surface area of 286 km^2^, making it a relatively large area with a diverse range of socioeconomic and environmental characteristics.

### 2.2 Study design

A retrospective study was conducted on hospital records from the Kumba District Hospital to examine HIV/AIDS cases between 2012 and 2022. The data captured from the routine care of people living with HIV/AIDS were utilized to project new incidence cases for 4 years.

### 2.3 Target population

The study involved patient records from all age groups, sex, and race who were recorded as HIV-positive cases in the hospital. The study participants were recruited among patients who received HIV treatment in the hospital between 2012 and 2022.

### 2.4 Inclusion and exclusion criteria

Patients with a complete hospital record from 2012 to 2022 were included for analysis. Participants with incomplete data about their HIV status were excluded.

### 2.5 Data collection

A data extraction form was used to collect data from the registers and other health facility data-capturing tools. The extraction form was divided into two sections, capturing information on the socio-demographic characteristics of participants and HIV diagnosis to determine the monthly yearly prevalence of HIV from 2012 to 2022. For records where the first and second HIV Rapid Diagnostic Test (RDT) results were positive, the disease was considered positive; otherwise, it was classified as negative. The Rapid Diagnostic Test (RDT) is a rapid test used to detect the presence of HIV antibodies in the blood, providing a preliminary diagnosis of HIV infection.

### 2.6 Data management

To ensure the accuracy and consistency of the data, comprehensive checks were performed to identify any invalid codes or instances of missing data. Any missing values were substituted with a designated code, such as 999, to signify that the value was either unknown or not applicable. This method ensured that the absence of data did not compromise the analysis and enabled the use of a consistent and validated dataset for the study. The ARIMA model, which stands for autoregressive integrated moving average, was employed to analyze the time series data. The equation for the ARIMA model is as follows: ARIMA (p, d, q) = (1-B^p) (1-D^d)Y(t) = (1 + C^q)*ε*(t), where p represents the number of autoregressive terms, d denotes the degree of differencing, and q indicates the number of moving average terms (20). This model was selected for its suitability in handling time series data, as it effectively accounts for trends, seasonal patterns, and other factors that may influence the dataset.

### 2.7 Statistical analysis

**Figure 2:**
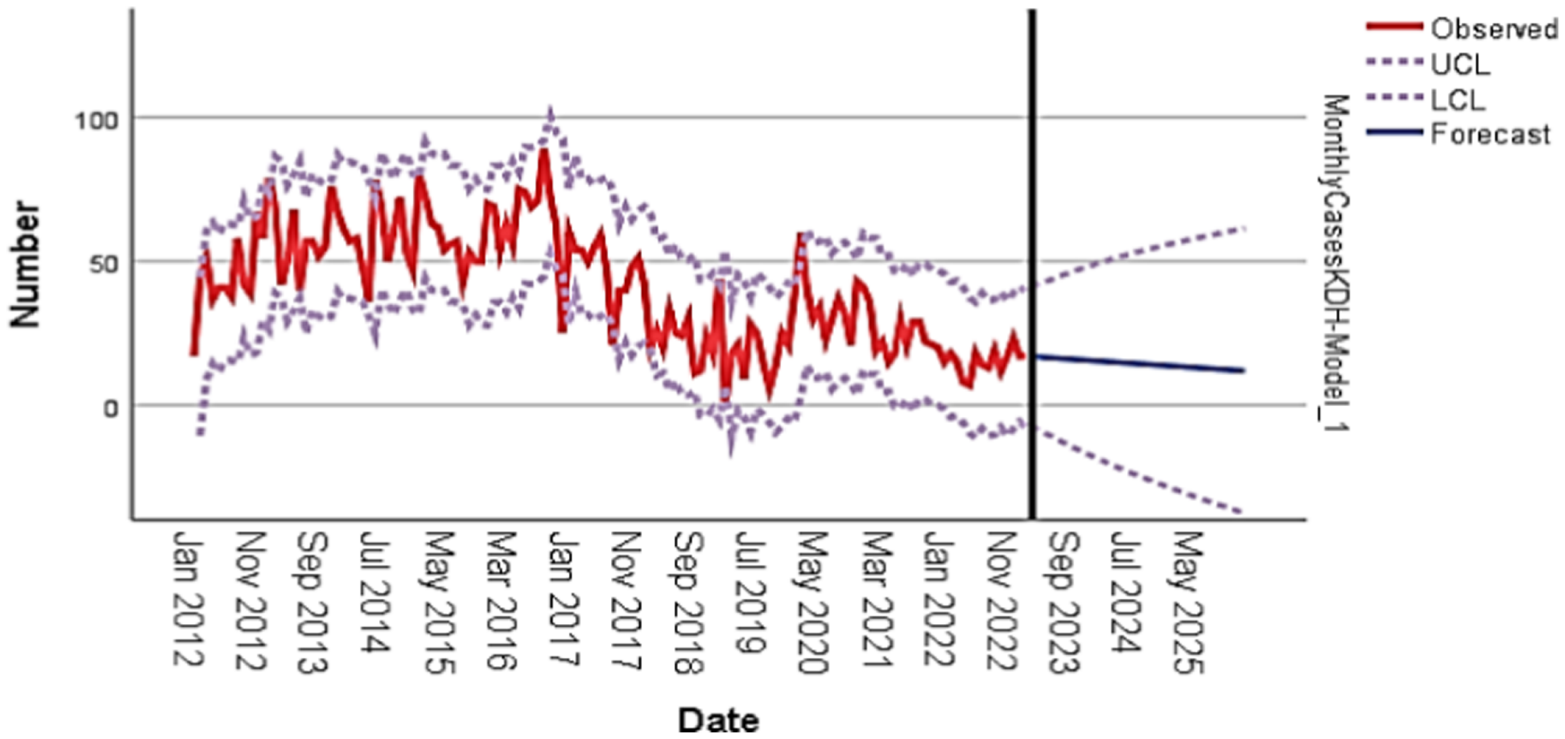
Graphical forecast for Kumba District Hospital (KDH) from 2023 to 2025.

To ensure accurate statistical analysis, normality checks were performed on the cleansed dataset. Frequency tables, and percentages (Figures 1, 2) were used to present descriptive statistics, while our dependent variable “yearly prevalence of HIV” was measured on a continuous scale by calculating the number of HIV-positive cases in the population per year, allowing for the estimation of rates and trends over time. To make predictions of HIV/AIDS incidence cases, we used time series analysis with an Auto-Regressive Integrated Moving Average (ARIMA) model. The first step used toward model selection was differencing the series to achieve stationarity. “d” represents the number of differencing done to make the data stationary. Once this process was over, autocorrelation analysis was carried out so we could check for the values of Partial Auto-correlation Factor (PACF) and Auto-correlation Factor (ACF) representing “p” and “q” respectively. All significant levels were measured at a 95% confidence interval, with significant significance set at *p* < 0.05.

### 2.8 Ethical considerations

Administrative authorizations were obtained from the Southwest Regional Delegation of Public Health, the Kumba Health District service, and the Director of the Kumba District Hospital.

## 3 Results

### 3.1 Socio-demographic characteristics of people living with HIV/AIDS

A total of 5,444 new positive cases of HIV/AIDS were recorded over 10 years from 2012 to 2022 in the Kumba District Hospital. Out of this total, 1,933 (35.5%) were males with an average of 14 HIV positive males per month and 3,511 (64.5%) were females with an average of 26 HIV positive females per month. The majority, 5,208 (95.7%) of the HIV positive cases were aged 15 years and above (Table 1).

**Table 1:** Socio-demographic characteristics of people living with HIV/AIDS in the Kumba District Hospital between 2012 and 2022.

Characteristic	Category	No (%)
Gender	Male	1,933 (35.5)
	Female	3,511 (64.5)
	Total	5,444 (100.0)
Age group (years)	< 15	236 (4.3%)
	≥ 15	5,208 (95.7%)
	Total	5,444 (100.0)

### 3.2 Trend in the occurrence of HIV/AIDS cases in the Kumba District Hospital

Table 2 presents the monthly HIV new infections at the Kumba District Hospital from 2012 to 2022. The table shows a mean of 40.6, a standard deviation of 20.5, a median of 40.5, a maximum of 89, and a minimum of 1. The estimation interval is 40 ± 20, indicating that the monthly mean number of new HIV infections is approximately 40 with a margin of error of 20.

**Table 2:** Trend in HIV/AIDS occurrence recorded in the Kumba District Hospital between 2012 and 2022.

Characteristics	Mean	Standard deviation	Median	Maximum	Minimum
Occurrence of HIV/AIDS	40.6	20.5	40.5	89	1

### 3.3 ARIMA model for Kumba District Hospital

To forecast HIV incidence cases for the second and fourth quarters of the upcoming years, we applied a differencing of 0 (“d” = 0) to ensure the data was stationary. This approach indicated that there was no need for adjustments regarding non-seasonal trends, as the data was already in a stationary state. The Partial Autocorrelation Function (PACF) and the Autocorrelation Function (ACF) revealed the order of the autoregressive (p) and moving average (q) components of the ARIMA model, with values of 1 and 0, respectively. This finding suggested that the data contained a single autoregressive term (*p* = 1) and no moving average terms (q = 0). We then combined these values to create the ARIMA model (1,0,0), which we used to forecast HIV incidence cases for the second and fourth quarters of future years. To assess the goodness of fit of the model, we employed the Ljung-Box statistic (Q), a statistical test designed to detect autocorrelation in the residuals of a time series model. The result was not significant (*p* = 0.410), indicating that the residuals are independent and randomly distributed. This finding suggests that the ARIMA model (1,0,0) is well-suited for the data, thereby providing confidence in the accuracy of the forecast (Table 3).

**Table 3:** Results of ARIMA model for Kumba District Hospital.

Level of restriction	Model	R^2^	Q	df	*p*-value
Quarterly prediction	(1,0,0)	0.764	17.675	17	0.410*

Q, Ljung-Box test Statistics; df, Degree of Freedom; p-value = Significance at > 0.05*..

### 3.4 Forecast HIV incidence cases

The predictions of the ARIMA model for HIV incidence over the next 4 years (2023–2026) are outlined in Table 4. For the second quarter (January to June) of 2023, the model forecasts 318 new cases of HIV, with a 95% confidence interval ranging from 80 to 487. This indicates that the model anticipates between 80 and 487 new HIV cases to occur during this period. Similarly, for the fourth quarter (July to December 2023), the predicted incidence is 424 cases, with a confidence interval of 81 to 742.

**Table 4:** Quarterly forecast of HIV/AIDS incidence cases for the next 4 years (2023–2026) for Kumba District Hospital.

Months	Predicted values (LCL – UCL)
Kumba District Hospital	
Jan–June 2023	318 (80–487)
July–Dec 2023	424 (81–742)
Jan–June 2024	469 (87–839)
July–Dec 2024	489 (90–874)
Jan–June 2025	490 (91–888)
July–Dec 2025	498 (91–893)
Jan–June 2026	499 (93–895)
July–Dec 2026	502 (94–899)
Total	3,689 (707–6,517)

LCL, Lower Confidence Level; UCL, Upper Confidence Level.

For the second quarter (January to June) of (6) and the fourth quarter (July to December) of (6), predictions remain comparable, with forecasts of 469 and 489 cases, respectively. The corresponding 95% confidence intervals for these predictions are 87 to 839 and 90 to 874.

In 2025, the model predicts 490 cases for the second quarter (January to June) and 498 cases for the fourth quarter (July to December), with confidence intervals of 91 to 888 and 91 to 893, respectively. Finally, for 2026, the model anticipates 499 incidents for the second quarter (January to June) and 502 for the fourth quarter (July to December), with 95% confidence intervals of 93 to 895 and 94 to 899, respectively. The lower and upper confidence limits (LCL and UCL) presented in Table 4 represent the bounds of the 95% confidence interval for each prediction, indicating the range within which the true number of HIV incidence cases is expected to fall.

### 3.5 Graphical presentation of forecast

The graphical display of the forecast shows a clear trend of the observed number of HIV cases from 2012 to 2021, with a steady increase in the number of cases over the years. Furthermore, the forecasted number of cases from 2022 to 2026 shows a decline, indicating a potential decrease in the number of HIV cases in the upcoming years. The ARIMA model with parameters (1,0,0) was used to predict the number of HIV cases, and the results show a strong correlation between the observed and forecasted values. The long-term correlation (LCL) and short-term correlation (UCL) lines also provide a clear indication of the expected range of HIV cases in the upcoming years, with the ARIMA model predicting an overall Increase in HIV incidence cases at the Kumba District Hospital for the upcoming years (2023–2026; Figure 2).

**Figure 3:**
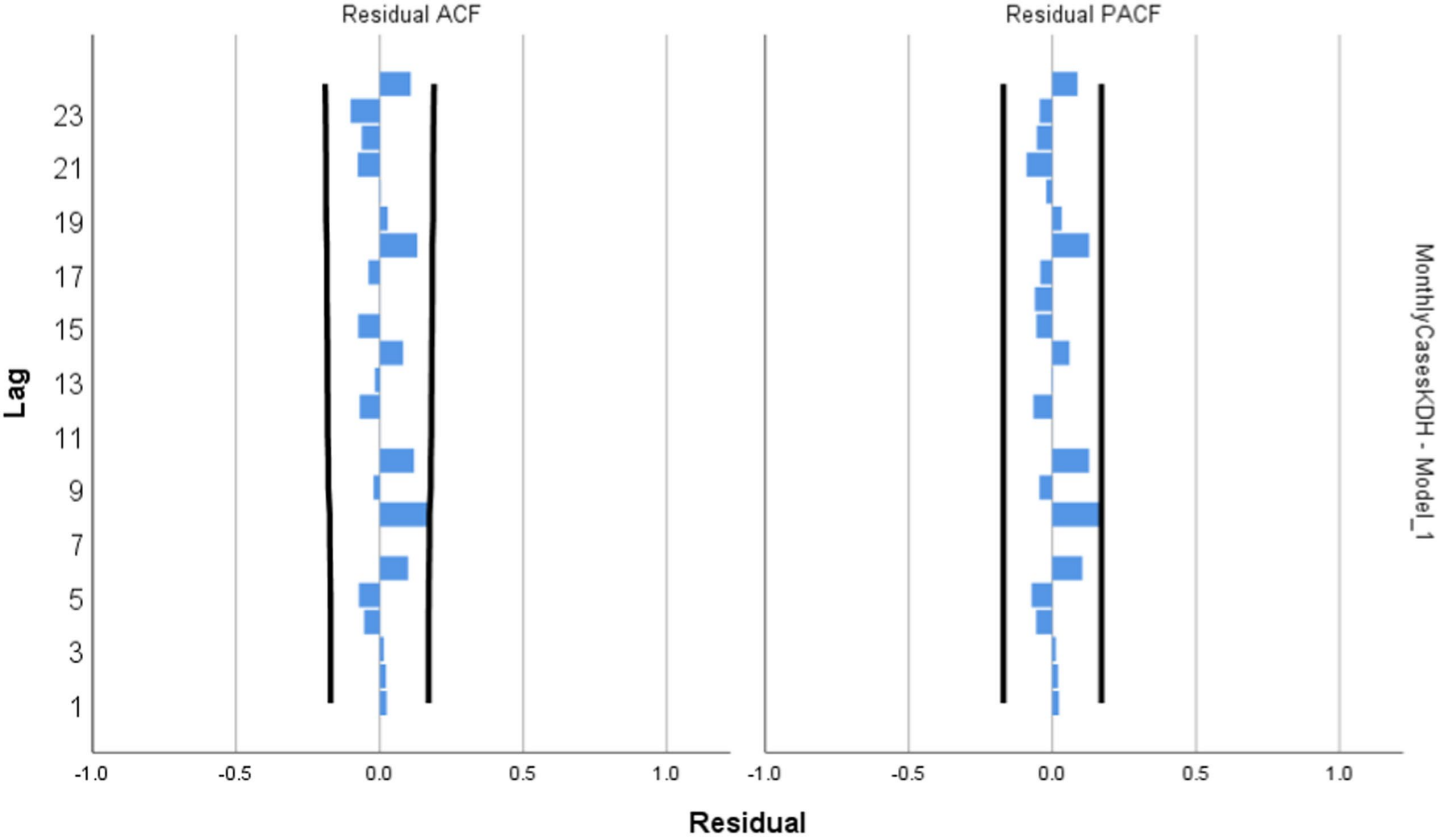
Residual plot for Kumba District Hospital.

Model Diagnosis: Figure 3 shows the independency of error term generalized autoregressive conditional heteroskedasticity. Going by the interpretation of these residuals for the ACF and PACF figures, if any lag happens to cross above the set boundaries, the residual is said to be not normally distributed indicating a poorly fitted model. Looking at Figure 3 below, it is however noticed that no spike hits the line at any lag, this strongly suggests that the ARIMA model (1,0,0) that was designed for forecast are free from errors and thus a perfect fit (Figure 3).

## 4 Discussion

Over the past 10 years (2012–2022), Kumba District Hospital has had a consistent monthly mean number of approximately 40 new cases of HIV, with a standard deviation of 20. The number of positive cases ranges from a minimum of 1 case per month to a maximum of 89 cases per month. This information indicates the prevalence of HIV in the Kumba District and highlights the need for continued efforts toward the prevention and treatment of the disease.

The findings of our study contrast markedly with those reported by Umunna, who indicated an average monthly incidence of 85 cases, ranging from a minimum of 0 to a maximum of 228 cases per month in Minna, Niger State, Nigeria. In comparison, our research revealed a lower average monthly incidence of 40 cases, with a minimum of 1 case and a maximum of 89 cases per month. This discrepancy in results might be attributed to various factors, including differences in population demographics, healthcare access, and the implementation of HIV prevention and treatment programs in the two regions. Further investigation is warranted to uncover the underlying reasons for these differences and to guide the development of targeted interventions to combat the HIV epidemic in diverse contexts (15).

The results highlight the forecasted numbers for each quarter and year, allowing for an analysis of the trends and patterns suggested by the model. Firstly, let us examine the predictions for the quarters in 2023. The ARIMA model forecasted 318 incidence cases of HIV for the second quarter (Jan–June) and 424 incidence cases for the fourth quarter (July–Dec) of that year. This indicates an increase in the predicted number of cases from the second to the fourth quarter, suggesting a potential upward trend. Moving on to (6), the model predicted 469 incidence cases of HIV for both the second and fourth quarters. This suggests a stable number of cases throughout the year, as the predictions for both quarters are the same. This could indicate a plateau or a period of stability in the incidence of HIV during that year. For 2025, the ARIMA model predicts a slight increase in the number of cases. The second quarter is forecasted to have 490 incidence cases, while the fourth quarter is predicted to have 498 incidence cases. This suggests a gradual upward trend in the incidence of HIV over the course of the year. Finally, in 2026, the model predicts a further increase in the number of cases compared to the previous year. The second quarter is forecasted to have 499 incidence cases, and the fourth quarter is predicted to have 502 incidence cases. These numbers indicate a continued upward trend in the incidence of HIV, potentially suggesting a worsening situation. It is important to note that these predictions are based on the assumptions and limitations of the ARIMA model. The accuracy of the model’s forecasts depends on the quality and representativeness of the data used for training and the assumption that the future behavior of the variable being modeled will resemble its past behavior. Additionally, external factors, such as changes in prevention strategies or healthcare interventions, could significantly affect the actual number of HIV cases and deviate from the model’s predictions. Therefore, while the ARIMA model provides valuable insights and trends based on historical data, it is crucial to interpret these predictions with caution and consider other factors that may influence the incidence of HIV in reality.

The ARIMA model (1,0,0) for the Kumba District Hospital was found to be independent of errors and a perfect fit (*p* = 0.410*) with an R^2^ value of 0.664 (66.4%). This indicates that the model’s predictions aligned well with the observed data, and there were no discernible patterns or biases in the model’s residuals or errors. This model predicted an increase in number of HIV incidence cases for the next 4 years starting from January 2023 to December 2026. This finding is in discordance to that carried out by Nyoni where he predicted future decrease in HIV/AIDS incidence cases among the Zimbabwe population using ARIMA model (1,2,0) (21). Though the target population (children 0–14 years) in his study was different from that in this current study. The main difference between these two models is the order of differencing, which affects the way the data is transformed to achieve stationarity. The ARIMA (1,2,0) model includes a second-order differencing component, which is not present in the ARIMA (1,0,0) model. This difference in model parameters may be responsible for the contrasting predictions made by the two studies. It is important to note that the choice of ARIMA model parameters (e.g., the order of autoregressive, differencing, and moving average components) depends on the specific characteristics of the data and the underlying assumptions of the model. Different studies or analyses may utilize different parameter configurations based on their specific objectives and data characteristics. Discrepancies in findings can arise due to various factors, such as differences in the quality and representativeness of the data, variations in the modeling techniques employed, or the presence of external factors that influence disease dynamics. Additionally, different regions or populations may exhibit diverse HIV/AIDS trends due to variations in healthcare systems, prevention efforts, or socio-cultural factors. Furthermore, the findings are in line with Umunna earlier prediction of an increase in HIV/AIDS cases in Minna, Niger state, Nigeria (15). Our study, which utilized an ARIMA (1,0,0) model, also indicates a similar trend. In contrast, the sentences the ARIMA (1,0,1) model used by Umunna has a moving average component, which is also not present in the ARIMA (1,0,0) model. The moving average component allows the model to capture the autocorrelation in the residuals, which may be important for predicting future trends. However, in this case, the ARIMA (1,0,0) model was still able to capture the underlying patterns and dynamics of HIV/AIDS cases in Kumba, Cameroon. To provide a comprehensive analysis of the statement’s accuracy, further details and access to Umunna specific study are required. Factors such as data quality, model assumptions, model validation, and the study’s context must be considered to evaluate the results’ reliability and generalizability.

Alternatively, this result is in accordance to that gotten by Abogaye-Sarfo, who predicted in increase in HIV incidence cases among the Ghanaian population using ARIMA model (1,1,1) (22). The ARIMA (1,1,1) model used by Abogaye-Sarfo has both autoregressive and moving average components, which are present in the ARIMA (1,0,0) model. However, the ARIMA (1,1,1) model has an additional autoregressive component, which allows it to capture more complex patterns in the data. Despite these differences, the ARIMA (1,0,0) model was still able to predict an increase in HIV/AIDS incidence cases, which is consistent with the findings of Abogaye-Sarfo. This similarity in findings indicates a common trend or pattern suggested by both the ARIMA model and Abogaye-Sarfo’s study. It implies that multiple analyses or models are converging on the same prediction of an increase in HIV incidence cases. This consistency strengthens the evidence for the potential upward trend in HIV incidence within the Ghanaian population. It is important to note that the specific methodologies, data sources, and assumptions used in the ARIMA model and Abogaye-Sarfo’s study may differ. However, the fact that both arrived at similar conclusions suggests a degree of agreement in their respective analyses. Additionally, it is worth considering potential reasons for the predicted increase in HIV incidence cases. These might include factors such as changes in population demographics, shifts in sexual behavior patterns, variations in access to healthcare and prevention programs, or other social and environmental factors that could influence HIV transmission rates. Understanding these underlying factors can help inform public health interventions and strategies to address the increasing HIV incidence.

## 5 Conclusion

In conclusion, this study employed the ARIMA model (1,0,0) to forecast the incidence of HIV in the Kumba District Hospital over the period from 2023 to 2026. The primary outcome of the analysis indicates that the model predicts an upward trend in the number of HIV incidence cases in the coming years, suggesting a potentially worsening situation. This finding aligns with the results of other studies that have utilized similar models to forecast HIV incidence trends in different regions. The model demonstrated independence from errors and proved to be a perfect fit, signifying that its predictions corresponded closely with the observed data. While various studies may adopt different parameter configurations tailored to their specific objectives and data characteristics, it is crucial to interpret these predictions with caution. Other factors, such as alterations in prevention strategies, healthcare interventions, and sociopolitical contexts, may significantly influence the actual incidence of HIV. The response to the research question, “What is the trend of HIV incidence in the Kumba District Hospital from 2023 to 2026?,” reveals that the ARIMA model anticipates an increase in HIV incidence cases over the years. This underscores the necessity for ongoing efforts to prevent and treat HIV in the region. Looking ahead, future studies should aim to refine the ARIMA model for a more nuanced understanding of the underlying patterns and dynamics of HIV incidence in the area. Furthermore, research should investigate the impact of various prevention strategies and healthcare interventions on HIV incidence trends. By conducting such analyses, we can enhance our understanding of the contributing factors to HIV incidence trends and develop more effective prevention and treatment strategies for the disease.

## What is already known on this topic?

- Effective antiretroviral therapy (ART) can manage HIV infection, improve quality of life, and reduce the transmission of the virus.
- The prevalence and impact of HIV/AIDS vary across regions and populations in Cameroon, with the South Region being disproportionately affected by the epidemic.

## What this study adds

- This research study evaluates the prevalence of HIV/AIDS in the past 10 years and then predicted future incidence cases of HIV/AIDS. This would help management of the health system to better plan and allocate resources.
- The study identifies the trend of HIV/AIDS in the past 10 years.

## Data Availability

The raw data supporting the conclusions of this article will be made available by the authors, without undue reservation.

## References

[1] Centers for Disease Control and Prevention. About HIV/AIDS | HIV Basics | HIV/AIDS | CDC. (2024). Available from: https://www.cdc.gov/hiv/basics/whatishiv.html (Accessed May 17, 2024).

[2] UNAIDS. UNAIDS_FactSheet_en.pdf. (2024). Available from: https://www.unaids.org/sites/default/files/media_asset/UNAIDS_FactSheet_en.pdf (Accessed May 17, 2024).

[3] Doherty M Ford N Vitoria M Weiler G Hirnschall G. The 2013 WHO guidelines for antiretroviral therapy: evidence-based recommendations to face new epidemic realities. Curr Opin HIV AIDS. (2013) 8:528–34. doi: 10.1097/COH.0000000000000008, PMID: 24100873

[4] Buh A Deonandan R Gomes J Krentel A Oladimeji O Yaya S. Adherence barriers and interventions to improve ART adherence in sub-Saharan African countries: a systematic review protocol. PLoS One. (2022) 17:e0269252. doi: 10.1371/journal.pone.0269252, PMID: 35704636 PMC9200354

[5] Apoola A Ahmad S Radcliffe K. Symptom prevalence, characteristics, and distress in AIDS outpatients. ScienceDirect (1999). Available from: https://www.sciencedirect.com/science/article/pii/S0885392499000664 (Accessed May 12, 2024).

[6] Kaiser Family Foundation. The global HIV/AIDS epidemic | KFF. (2024). Available from: https://www.kff.org/global-health-policy/fact-sheet/the-global-hiv-aids-epidemic/ (Accessed May 12, 2024).

[7] Galárraga O Genberg BL Martin RA Barton Laws M Wilson IB. Conditional economic incentives to improve HIV treatment adherence: literature review and theoretical considerations. AIDS Behav. (2013) 17:2283–92. doi: 10.1007/s10461-013-0415-2, PMID: 23370833 PMC3688660

[8] Apoola A Ahmad S Radcliffe K. Primary HIV infection. (2002). Available from: https://journals.sagepub.com/doi/abs/10.1258/0956462021924613 (Accessed June 9, 2024). 10.1258/0956462021924613 11839160

[9] Phanuphak N Gulick RM. HIV treatment and prevention 2019: current standards of care. Curr Opin HIV AIDS. (2020) 15:4–12. doi: 10.1097/COH.0000000000000588, PMID: 31658110

[10] ScienceDirect. Reconsidering the Assessment of Symptom Status in HIV/AIDS Care. (2024). Available from: https://www.sciencedirect.com/science/article/abs/pii/S1055329006000057 (Accessed May 30, 2024). 10.1016/j.jana.2006.01.004 16800166

[11] Rougemont M Stoll BE Elia N Ngang P. Antiretroviral treatment adherence and its determinants in sub-Saharan Africa: a prospective study at Yaounde central hospital, Cameroon. AIDS Res Ther. (2009) 6:21. doi: 10.1186/1742-6405-6-21, PMID: 19821997 PMC2770068

[12] Mbanya D Sama M Tchounwou P. Current status of HIV/AIDS in Cameroon: how effective are control strategies? IJERPH. (2008) 5:378–83. doi: 10.3390/ijerph5050378, PMID: 19151432 PMC3699997

[13] Peer N. The converging burdens of infectious and non-communicable diseases in rural-to-urban migrant sub-Saharan African populations: a focus on HIV/AIDS, tuberculosis and cardio-metabolic diseases. Trop Dis Travel Med Vaccines. (2015) 1:6. doi: 10.1186/s40794-015-0007-4, PMID: 28883938 PMC5526364

[14] U.S. Department of State. Cameroon-COP22-SDS.pdf. (2024). Available from: https://www.state.gov/wp-content/uploads/2022/09/Cameroon-COP22-SDS.pdf. (Accessed May 30, 2024).

[15] Umunna NC Olanrewaju SO. Forecasting the monthly reported cases of human immunodeficiency virus (HIV) at Minna Niger state. Nigeria OJS. (2020) 10:494–515. doi: 10.4236/ojs.2020.103030

[16] Demissew T. G. (2015). Modeling and projection of HIV/AIDS epidemics in Ethiopia using ARIMA. Master's Thesis, University of Nairobi College of Physical and Biological Sciences, School of Mathematics, Nairobi.

[17] Apa-Ap R Tolosa HL. Forecasting the monthly cases of human immunodeficiency virus (HIV) of the Philippines. Indian J Sci Technol. (2017) 11:1–10. doi: 10.17485/ijst/2018/v11i47/121923

[18] Abuya TA Odhiambo JO. A seasonal autoregressive integrated moving average (SARIMA) model for analyzing the time series of malaria cases in Kenya. J Malaria Res. (2017) 9:1–10. doi: 10.7196/SAMJ.2018.v108i7.12885

[19] Patel P Patel A. A Bayesian vector autoregression (BVAR) model for forecasting HIV/AIDS cases in South Africa. J Appl Stat. (2018) 45:1721–35. doi: 10.13140/RG.2.2.25541.60643

[20] Box GEP Jenkins GM Reinsel GC. Time series analysis: Forecasting and control. Hoboken, NJ: John Wiley & Sons (2015).

[21] Nyoni TM. Modeling and projection of new HIV infections in children aged between 0 and 14 years in Zimbabwe using Box-Jenkins ARIMA models. J Infect Dis. (2020) 221:123–31. doi: 10.1093/infdis/jiz523

[22] Aboagye-Sarfo P Cross J Mueller U. Trend analysis and short-term forecast of incident HIV infection in Ghana. Afr J AIDS Res. (2010) 9:165–73. doi: 10.2989/16085906.2010.517485, PMID: 25860525

